# Infantile Spasms: Clinical profile and treatment outcomes

**DOI:** 10.12669/pjms.346.15869

**Published:** 2018

**Authors:** Shazia Kulsoom, Shahnaz H Ibrahim, Sidra Kaleem Jafri, Khemchand N Moorani, Misbah Anjum

**Affiliations:** 1 *Dr. Shazia Kulsoom, MBBS, FCPS, Department of Pediatrics and Child Health, Aga Khan University Hospital, Karachi, Pakistan*; 2 *Dr. Shahnaz H Ibrahim, Professor of Pediatrics and Pediatric Neurologist, Department of Pediatrics and Child Health, Aga Khan University Hospital, Karachi, Pakistan*; 3 *Dr. Sidra Kaleem Jafri, MBBS, FCPS., Department of Pediatrics and Child Health, Aga Khan University Hospital, Karachi, Pakistan*; 4 *Dr. Khemchand N Moorani, Professor of Pediatrics and Pediatric Nephrologist, NICH. Department of Pediatric Medicine Unit III, National Institute of Child Health, Karachi, Pakistan*; 5 *Dr. Misbah Anjum, MBBS, FCPS. Department of Pediatric Medicine Unit III, National Institute of Child Health, Karachi, Pakistan*

**Keywords:** Epileptic Encephalopathies, Electroencephalographic, Hypsarrythmia, Myoclonic jerks, Vigabatrin

## Abstract

**Background and Objective:**

Infantile spasm (IS) is one of the severe epileptic encephalopathies which affect children in early two years of life. Our objective was to determine the clinical profile, etiology and outcome of treatment in children with infantile spasms attending tertiary care hospital at Karachi, Pakistan.

**Methods:**

This is retrospective study of 36 patients out of 94 registered as IS, aged three months to two years, managed and followed up at Aga Khan University Hospital, Karachi, from 2010 to 2015. Data of all children with IS was collected from case record. Details including clinical observations, lab investigations, anti-epileptic medications and treatment outcome was collected and analyzed. Patients who received treatment for six weeks to document response were included. The treatment response was categorized as complete response, partial response (>50% improvement) and no response. Data was analyzed on SPSS using descriptive statistics.

**Results:**

Thirty- six patients (38.29%) with IS fulfilled eligibility criteria. The mean ± SD age at presentation was 4.6±2.1 months. Male to female ratio was 2:1. Consanguinity and developmental motor delay was observed in 66.6% and 89% respectively. Symptomatic etiology was predominant (61%) and hypoxic ischemic insult (32%) was the commonest underlying cause. EEG and MRI were diagnostic tools whereas metabolic studies were not helpful. Multiple antiepileptic drugs were used for seizure control and vigabatrin was the most frequently used (88%) drug. Short term treatment response was not different in idiopathic or symptomatic infantile spasms.

**Conclusion:**

Majority of patients had symptomatic infantile spasms and generalized tonic clonic along with myoclonic jerks were predominant seizure types. EEG and MRI were diagnostic in most of cases. Multiple AEDs were required to control seizures and VGB was most common drug (88%) used. Treatment outcome was not different in idiopathic and symptomatic groups.

## INTRODUCTION

Infantile spasm (IS) is a distinctive disorder, which affects the individual during infancy and early childhood.^1^,^2^ It is one of the severe epilepsies of childhood and is indicative of a true Epileptic Encephalopathy.^3^ The incidence of infantile spasms is one per 2000-6000 live births.^4^,^5^ Infantile spasms usually develops before 12 months of age, with only 8% of cases occurring in children above one year of age.^6^

It is characterized by abrupt, symmetric flexion and/or extension of the neck, trunk and extremities. IS is diagnosed by typical electroencephalographic (EEG) findings of hypsarrythmia.^7^ Infantile spasms have been classified as symptomatic when etiology is known and cryptogenic/idiopathic when no known cause is found. Majority (60-80%) of the cases represent symptomatic group.^8^

The type of convulsion in IS depends on which muscle groups (flexor or extensor) are primarily affected and on the extent of the contraction. Flexor spasms have long been considered as the most characteristic type of seizure constituting 42% of the cases while mixed flexor extensor spasms form 50% of all cases.^9^

The etiology is highly varied and includes neurocutaneous syndromes, metabolic disorders, cortical malformations, perinatal brain injuries, postnatal infections and head trauma. Commonly, infantile spasms are associated with hypoxic-ischemic insult, tuberous sclerosis and cortical malformations.^10^ Children may present with infantile spasms alone or in combinations of West syndrome. West syndrome is characterized by cognitive deterioration along with infantile spasms and hypsarrhythmia or modified hypsarrhythmia on EEG.^11^

Children with IS require extensive evaluation to identify the etiology and an EEG is essential to confirm the diagnosis of infantile spasm. Magnetic Resonance Imaging (MRI), metabolic workup and more recently genetic analysis may be required.^12^

Management of infantile spasm is challenging one since standard anticonvulsant drugs (AEDs) are not effective. Long term outcome is not well established and the children with IS often has a poor outcome. Disagreement regarding optimal treatment and long-term outcome exists due to variation in classification, treatment strategies and lack of evidence-based cost-effective AEDs. The outcome of IS based on etiology is different in symptomatic and cryptogenic /idiopathic group.^13^

Management of patients in resource-limited settings is constrained by delay in diagnosis and high cost of remedies.^14^ Though information exists on various aspects of childhood seizure disorders, but there is little information in the literature regarding infantile spasms in developing countries specially Pakistan.^2^ Henceforth, this retrospective study was conducted to evaluate the clinical spectrum and treatment outcome of children with infantile spasms at tertiary care center in a developing country.

## METHODS

This study was conducted at the Department of Pediatrics and Child Health, the Aga Khan University Hospital, a private sector tertiary care teaching hospital in Karachi, Pakistan. The study protocol was approved by Institutional Ethics Committee (4147-Ped-ERC-17). Data were collected by retrospective chart review of all patients diagnosed as Infantile Spasms from January 2010 to December 2015. Cases were identified by using hospital information management system and international classification of disease-10 (ICD-10) coding G40.82 for infantile spasms.

Patients were evaluated with detailed history, physical examination and pertinent investigations were carried out. Patients were diagnosed as IS when they presented with characteristic epileptic spasms and hypsarrhythmia or modified hypsarrythmia was found on EEG.

Further workup like MRI was done for associated risk factors (eg perinatal insult) or etiological factors (neurocutaneous syndrome, inborn errors of metabolism). Children with consanguineous parents, history of sibling deaths and developmentally delay in family members underwent metabolic workup like plasma ammonia, lactate, amino acids and urine for organic acids. Comorbid conditions like visual problems, hearing impairment and recurrent chest infection were assessed and recorded.

Based on the etiology, IS was classified as symptomatic (known etiology) or cryptogenic/ idiopathic (unknown etiology). Patients were treated initially with standard AEDs (e.g. phenobarbitone, valproic acid) and subsequently according to need, placed on specific drugs like oral vigabatrin (VGB), prednisolone and parenteral adrenocortical hormone (ACTH) or methylprednisolone. Outcome of the treatment was assessed at six weeks and categorized as complete response when no seizures observed after six weeks, partial response if more than 50% decrease in spasms and no response if patient remain symptomatic.

Children aged less than three months, aged more than two years and with follow -up for less than 12 months were excluded. The data collected on proforma included gender, age of onset of infantile spasms, age at diagnosis and initiation of treatment, type of spasms (flexor, extensor and others), etiological factors (perinatal events, mode of delivery, developmental delay, neurocutaneous syndromes), MRI and EEG findings as well type of treatment and response.

The data was analyzed by SPSS version 19 using descriptive statistics. Categorical variables like gender and response to treatment were expressed as frequency and percentages while continuous variables like age were expressed as mean+/- SD. Chi square test was used to see the association of etiology with treatment response and P value of <0.05 was taken as significant.

## RESULTS

A total of 94 patients with IS were registered during study period and 36 (38.29%) fulfilled the eligibility criteria. Clinical profile of study population is shown in Table-I. Boys were predominant (66. 6%). The mean ± SD age of onset of symptoms, diagnosis and initiation of treatment was 4.6*±* 2.1 and 6.5*±* 3.6. months respectively. Lag time from onset of symptom and diagnosis in our study was two months. Consanguinity and developmental motor delay were observed in 66.6% and 89% respectively. The most common type of epileptic spasm was generalized tonic clonic along with myoclonic jerks (38.7%) followed by flexor spasms (30.5%) and mixed type (28%).

**Table-I:** Clinical Profile of patients with Infantile Spasms (N=36).

Variable	Values
Gender	Male 24 (66.6%)
	Female 12(33.33%)
Cnsanguinity	26 (72%)
Microcephaly	24 (66.6%)
Developmental delay	32 (89%)
***Mode of delivery***	
LSCS	23 (64%)
SVD	13(36%
***Age (months)±SD***	
At onset of spasm	4.6±2.0
At diagnosis of spasm	6.5±3.5
At initiation of treatment	6.5±3.5
***Type of spasms***	
Flexor	11 (30.5%)
Extensor	01(2.8%)
Mixed	10 (28%)
Generalized tonic clonic along with myoclonus	14 (38.7%)
***Comorbidities***	
Visual impairment	6 (16.7%)
Hearing impairment	2 (5.6%)
Recurrent chest infections	7 (19.5%)
More than two comorbidities	13 (36%)
None	8(22.2%)

Forty two percent had weight below 5^th^ percentile and 61% were micro cephalic. Based on etiological classification (Table-II), symptomatic IS was the most common type (61%). The most common cause among the symptomatic infantile spasms was hypoxic ischemic insult (32%), followed by post meningitic sequelae (13.6%) and perinatal stroke (13.6%). Two children had IEM and one had tuberous sclerosis.

**Table-II:** Etiological Profile of children with Symptomatic Infantile Spasms (N=22).

Etiology	N (%)
Hypoxic ischemic encephalopathy	7 (32)
Post-meningitic sequalae	3 (13.6)
Congenital brain malformation	3 (13.6)
Perinatal stroke	3 (13.6)
Hypoglycemia	2 (9)
Inborn errors of metabolism	2 (9)
Tuberous sclerosis	1 (4.6)
Prematurity	1 (4.6)

The diagnostic workup in children with IS is shown in Table-III. Hypsarrhythmia (69%) was the most common EEG finding followed by modified hypsarrhythmia (31%). Fifty two percent of our patients underwent repeat EEG after six weeks of therapy and it was normal in 32% patients.

**Table-III:** Diagnostic Profile of children with Infantile Spasm (N=36).

Electrophysiological studies	N (%)
Hypsarrhythmia	25 (69)
Modified hypsarrhythmia	11(31)
***Magnetic Resonance Imaging findings***	*N= 36*
Normal	23 (64)
Abnormal MRI findings	13 (36)
Ischemia/hemorrhage	6 (46)
Congenital brain malformation	3 (23)
Brain atrophy	2 (15.6)
Cortical tubers	1 (7.7)
Brain tumor	1 (7.67)
Metabolic workup	25 (70%)
Biotinidase deficiency	1 (4)
Elevated VLCFA	1 (4)

VLCFA= Very long chain fatty acids

MRI was carried out in all patients; it was normal in 64% and abnormal in 36%. Most common radiological findings were vascular events (ischemia/hemorrhage in 46%) followed by congenital brain malformations (23%). Metabolic workup was carried out in 25 children (70%) and it showed biotinidase deficiency and elevated very long chain fatty acids each in one case. All children received multiple AEDs and 52% of cases required more than four AEDs to control the seizure. VGB was used in 88% of the children while ACTH was given to 50%.

Use of VGB preceded ACTH except for two children in whom ACTH was used without VGB, because one was already on ACTH replacement therapy for panhypopituitarism and in other VGB was contraindicated.

Considering the treatment response (Table-IV), complete response was almost similar in both groups (symptomatic 27% vs cryptogenic 28%) whereas partial response (50% vs 64%) and no response (22.8% vs 7.1%) were different in cryptogenic/ idiopathic and symptomatic group but it was not significant (p-value 0.41). Hypertension was observed in 50% of children who received ACTH whereas only one developed cushingoid appearance.

**Table-IV:** Therapeutic Response in Children with Infantile Spasms (N=36).

Response to treatment	Symptomatic group N=22(61%)	Idiopathic group N=14(39%)
Complete**** response*	6 (27.2)	4 (28.6)
Partial response *	11 (50)	9(64.3)
No response	5 (22.8)	1(7.1)

* P-value =0.41

## DISCUSSION

This study highlights the clinical profile and outcome of children with infantile spasm at a tertiary care center with well-established pediatric neurology subspecialty. We observed a male preponderance which is similar to other studies from India, Sweden and South Africa.^15^,^16^

The mean age of onset of infantile spasms in our study (4.6 months) is comparable to a study by Kaushik JS et al. in which it was 5.3 months.^15^ Mean lag time in our study (2 months) is less than reported by Kaushik JS et al.^15^ However, it is much less in developed countries (25- 45 days).^13^,^17^ This may reflect lack of awareness of this condition in parents, family physicians and general pediatricians.^18^ Furthermore, this paucity of knowledge may have resulted in delay in diagnosis, inappropriate choice and dosage of AEDs.^19^,^20^

In the current study, we found symptomatic epileptic spasms in 61% which is similar to our previous study from same center (64.3%). Among the symptomatic group, perinatal events constituted 32% which has significantly decreased from previous figures (70%).^21^ This may be due to improvement in availability and better obstetric care.^22^ A similar recent study, published from South Africa by Keshave et al showed that perinatal events accounted for 50% of the symptomatic cases.^23^

The most common type of epileptic spasm in our study was flexor spasms (30.5%) which is less than 76% reported by Kaushik JS et al.^15^ Mixed type of spasms (28%) in our study is more than reported by Kaushik JS et al. We found hypsarrythmia pattern in EEG, the most common occurrence (69%) followed by modified hypsarrythmia (31%) which is well established findings in many studies.^15^,^22^-^24^ Keshave et al. reported hypsarrythmia in 62% of patients in his study.^23^

The primary goal of management in infantile spasms is seizure control and improved long term developmental outcome. Though we did not find difference in treatment response in symptomatic versus idiopathic IS groups, but overall response was better in cryptogenic/idiopathic (92.9%) compared to symptomatic spasms (77.2%). Similar treatment response has been reported in other studies.^1^,^22^,^23^,^25^

### Strengths and Limitations

The study on clinical profile and treatment outcome of children with IS over a period of 5 years with complete follow up highlights the pediatric neurology practice. However, we acknowledge our limitation of a small sample size from a single center in which we have not considered detailed response to individual AEDs.

## CONCLUSION

In our study symptomatic infantile spasms were predominant and were associated with consanguinity (66.6%), developmental delay (89%) and microcephaly. Generalized tonic -clonic seizure along with myoclonic jerks (38.7%), flexor spasms (30.5%) and in combination (28%) were type of seizures. Hypoxic ischemic insult (32%) was important cause of symptomatic infantile spasms. EEG was diagnostic in most of cases; MRI was useful in detecting vascular events and brain malformations whereas metabolic workup was not helpful. Multiple AEDs were required to control seizures and VGB was most common drug (88%) used. Short term treatment response was not different in idiopathic or symptomatic infantile spasms.

This may help pediatricians in identifying types of infantile spasms, guiding diagnostic workup and treatment outcome. Studies with larger sample size and prospective in nature to determine the outcome are suggested.

### Author’s Contribution

**SK:** Conceived ideas, data collection and prepared manuscript.

**SHI:** Guided and edited manuscript.

**SKJ:** Did statistical analysis.

**KNM:** Reviewed for final approval of manuscript.

**MA:** Did write-up and literature search.
